# Investigating the role of circulating CXCR5-expressing CD8+ T-cells as a biomarker for bacterial infection in subjects with pneumonia

**DOI:** 10.1186/s12931-019-1011-4

**Published:** 2019-03-12

**Authors:** Yu Shen, Qiu-xia Qu, Meng-ni Jin, Cheng Chen

**Affiliations:** 1 Clinical Immunology Laboratory, the First Affiliated Hospital of Soochow University, 188 Shizi Street, Suzhou, 215006 China; 2 Jiangsu Key Laboratory of Clinical Immunology, Soochow University, 708 Renmin Road, Suzhou, 215006 China; 3 Respiratory Department, the First Affiliated Hospital of Soochow University, 188 Shizi Street, Suzhou, 215006 China

**Keywords:** CXCR5, CD8+ T cell, Bacterial infection, Pneumonia, PCT

## Abstract

**Background:**

Recently, lymphoid follicle-confined and circulating CD8+ T-cells expressing the C-X-C chemokine receptor type 5 (CXCR5) were described, which was involved in anti-virus immune response. However, the dynamics and role of circulating CXCR5-expressing CD8+ T-cells during bacterial infection is unknown. So, we asked whether CXCR5+ CD8+ T cells were also generated during bacterial infections in lower respiratory tract.

**Methods:**

The clinical data of 65 pneumonia patients were analyzed. The patients were divided into groups as tuberculosis, bronchiectasis and community or hospital acquired pneumonia (CAP, HAP). The sputum/bronchial secretion or bronchoalveolar lavage fluid (BALF) samples were taken for microbiological examination. The procalcitonin (PCT) was used to evaluate disease severity of these groups and compared among patients. We characterized the number and phenotype (PD-1 and CD103) of CXCR5 + CD8+ T cells in the peripheral circulation by flow cytometry in all individuals and analyzed their association with the serum PCT level and disease severity.

**Results:**

Patients were mainly infected with *Escherichia coli*, Acinetobacter baumannii, Klebsiella pneumonia (K.p), *Pseudomonas aeruginosa*, and *Staphylococcus aureus*. Of note is the finding that PCT was weakly correlated with severity of respiratory infections. Furthermore, it was revealed an increase of CXCR5-expressing CD8+ T cells in peripheral blood of un-controlled CAP and progressive HAP compared controlled CAP and HAP, respectively (*P* < 0.05). Strikingly, the circulating CXCR5-expressing CD8+ T-cells in K.p-infected group was higher than that non-K.p-infected group (*P* < 0.05). Meanwhile, the ratio of CXCR5 + CD8+/CD8 was positively correlated with PCT level (*P* < 0.05). In clinic, the determination of CXCR5-expressing CD8+ T-cells showed better results compared to PCT and can be useful for the prediction of exacerbation of CAP or HAP. Phenotypically, CXCR5+ CD8 + T cell expressed comparable level of inhibitory molecules PD-1 and lower CD103 compared to their CXCR5- counterparts.

**Conclusion:**

The circulating CXCR5-expressing CD8+ T-cell has diagnostic value for current pneumonia severity, and could act as a biomarker for identifying a bacteria-associated exacerbation. These cells may provide novel insight for the pathogenesis of pneumonia.

## Introduction

The coordinated differentiation of distinct effector populations of T cells is required for the efficient elimination of infected or cancerous cells. C-X-C chemokine receptor type 5 (CXCR5) is usually not expressed on CD8+ T cells, but it is required for the migration of follicular cytotoxic T cells (TFC) into B cell follicles. A population of CD8+ T-cells within lymphoid follicles was described as follicular CD8+ T-cells, characterized by the expression of CXCR5 and low or null expression of the C-C chemokine receptor type 7 (CCR7) [1]. This follicle confined CD8+ T-cell population exhibits lytic and nonlytic mechanisms and has been associated with viral infections control and cancer [2–4]. Interestingly, although the main localization of follicular T-cells are the secondary lymphoid organs, CXCR5-expressing T-cells are also found in peripheral blood [5]. Although it is clear that CXCR5 + CD8+ T cells was involved in anti-viral immune response, surprisingly little is known about it during bacterial infections in lower respiratory tract.

In clinical work, subjects with pneumonia can be diagnosed early by identifying pathogenic bacteria, detecting auxiliary inflammatory cytokines and observing clinical symptoms. Common laboratory examinations include blood routine, procalcitonin (PCT) [6, 7], white blood cell count (WBC), high-sensitivity C-reactive protein (hs-CRP) and neutrophil percentage (NEU%). Here, we characterized circulating CXCR5-expressing CD8+ T-cells in pneumonia individuals and analyzed their association with PCT level. A potential role of CXCR5+ CD8+ T-cells in the setting of exacerbation of CAP and HAP was suggested, especially K.p infection. In addition, the expression profile of PD-1 and CD103 by CXCR5 + CD8+ T-cells was associated with their immune phenotype.

## Patients and methods

### Study participants

In this study, 65 subjects with respiratory infection were included. The diagnosis of community acquired pneumonia (CAP) and hospital acquired pneumonia (HAP) was established according to the American Thoracic Society/European Respiratory Society (ATS/ERS) standards. From all 65 patients, 5 patients were classified as tuberculosis, 11 patients were classified as bronchiectasis, 23 and 26 patients were grouped in CAP and HAP respectively. Patients with chronic obstructive pulmonary disease (COPD), asthma, auto-immune disease and malignant disease were excluded from this investigation. We considered patients progressively according to the criteria: pneumonia with two or more complications, for example acute respiratory distress syndrome, heart failure, septic shock, and secondary infections, and no improvement of at least one of these complications after 3 days of active treatment [8, 9]. The study was approved by the Ethics Committee of our Institute of the First Affiliated Hospital of Soochow University.

### Flow cytometry analysis

5 mL of venous blood was drawn in an anti-coagulant tube from each subject. The following mixtures of antibodies were used for cell phenotype: anti-CD45- fluorescein isothiocyanate (FITC), anti-CD45-phycoerythrin-cyanine 7 (PE-CY7), anti-CD8-allophycocyanin (APC), anti-CD103-PE-CY7, anti-PD-1-FITC and anti-CXCR5-phycoerythrin (PE). For the detection of CD8+ cells, cells were incubated with anti-CD45 mAb and anti-CD8 mAb in the dark at room temperature for 30 min. The percentages of CXCR5-expressing CD8+ cells were estimated by comparing the proportions of labeled cells with respect to total number of CD8+ cells from the subjects studied. Cell surface antigen (PD-1, CD103) expression was assessed by plotting them versus the given CD8 + CXCR5+ cells. The samples were analyzed by a FACS Calibur flow cytometer.

### Laboratory testing

The sputum (*n* = 45) or bronchoalveolar lavage fluid (BAL, *n* = 20) samples were delivered for microbiological examination. All samples were inoculated on bacterial media. PCT was measured using B.R.A.H.M.S. PCT automated immunoassays. The analytical sensitivity of all assays was < 0.25 g/L. All techniques were based on a one-step immunoassay sandwich method.

### Statistical analysis

Statistical analysis was performed with SPSS statistical software (Version 19.0; SPSS Inc., Chicago, IL, USA). Student’s t test was used to compare the CXCR5 + CD8+/CD8+ T-cell ratio between studied groups. Two-sided Fisher exact test were applied to determine the strength of association between the categorical variables. Receiver operating characteristic (ROC) curves were calculated to select the cut-off level of ratio of CXCR5 + CD8+ T cells/CD8+ cells and PCT value indicating exacerbation of CAP and HAP. All tests were two sided with a *P*-value of less than 0.05 being considered statistically significant.

## Results

### Subject characteristics

A total of 65 eligible patients were enrolled. Baseline characteristics of the subjects were listed in Table 1. The patients include 53 men and 10 women and the median age was 63 years (range, 36–95 years). A total of 49 community acquired pneumonia (CAP) and hospital acquired pneumonia (HAP) individuals, 12 and 14 patients were controlled CAP and HAP respectively, 11 CAP and 12 HAP patients had disease progress.

**Table 1:** Subject characteristics

Type of disease	*n* = 65
Tuberculosis	5
Age (y)	19–63
Male	4
Female	1
Bronchiectasis	11
Age (y)	44–85
Male	8
Female	3
Non-K.p	7/12
K.P	0/12
Controlled CAP	12
Age (y)	16–95
Male	7
Female	5
Non-K.p	1/12
K.P	0/12
Progressive CAP	11
Age (y)	51–90
Male	8
Female	3
Non-K.p	3/11
K.P	3/11
Controlled HAP	14
Age (y)	20–90
Male	10
Female	4
Non-K.p	12/14
K.P	1/14
Progressive HAP	12
Age (y)	41–81
Male	10
Female	2
Non-K.p	4/12
K.P	7/12

### Circulating CXCR5^+^ CD8+ T-cells in acute, chronic and exacerbated bacterial infection

**Fig. 1:**
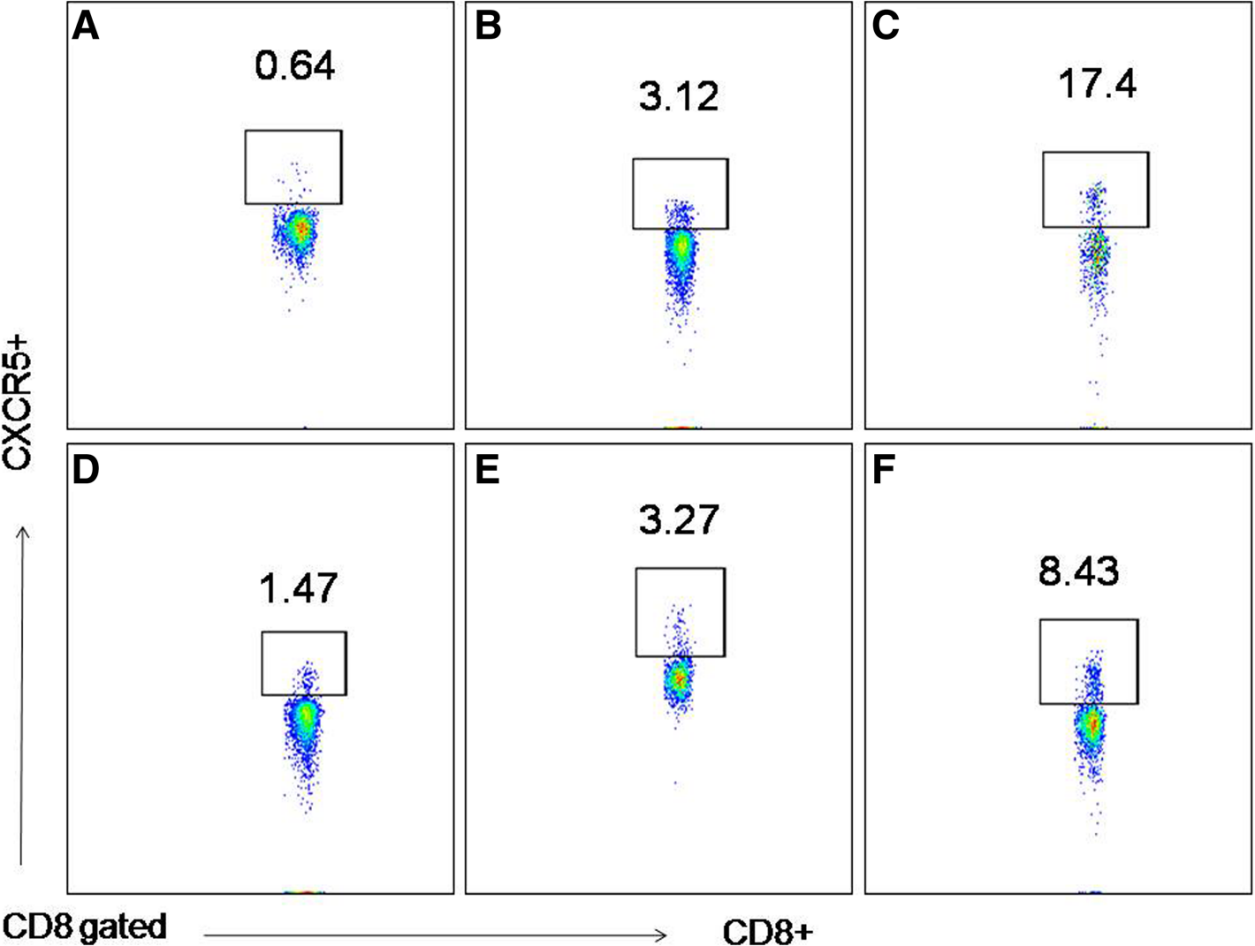
The ratio of circulating CXCR5-expressing CD8+ T cells among CD8 + T cells in all individuals was determined by flow cytometry. **a**: tuberculosis, **b**: controlled HAP, **c**: un-controlled HAP, **d**: bronchiectasis, **e**: controlled CAP, **f**: progressive CAP. Representative FACS plots of CXCR5 expression in CD8+ T cells from lymphocytes are shown

**Fig. 2:**
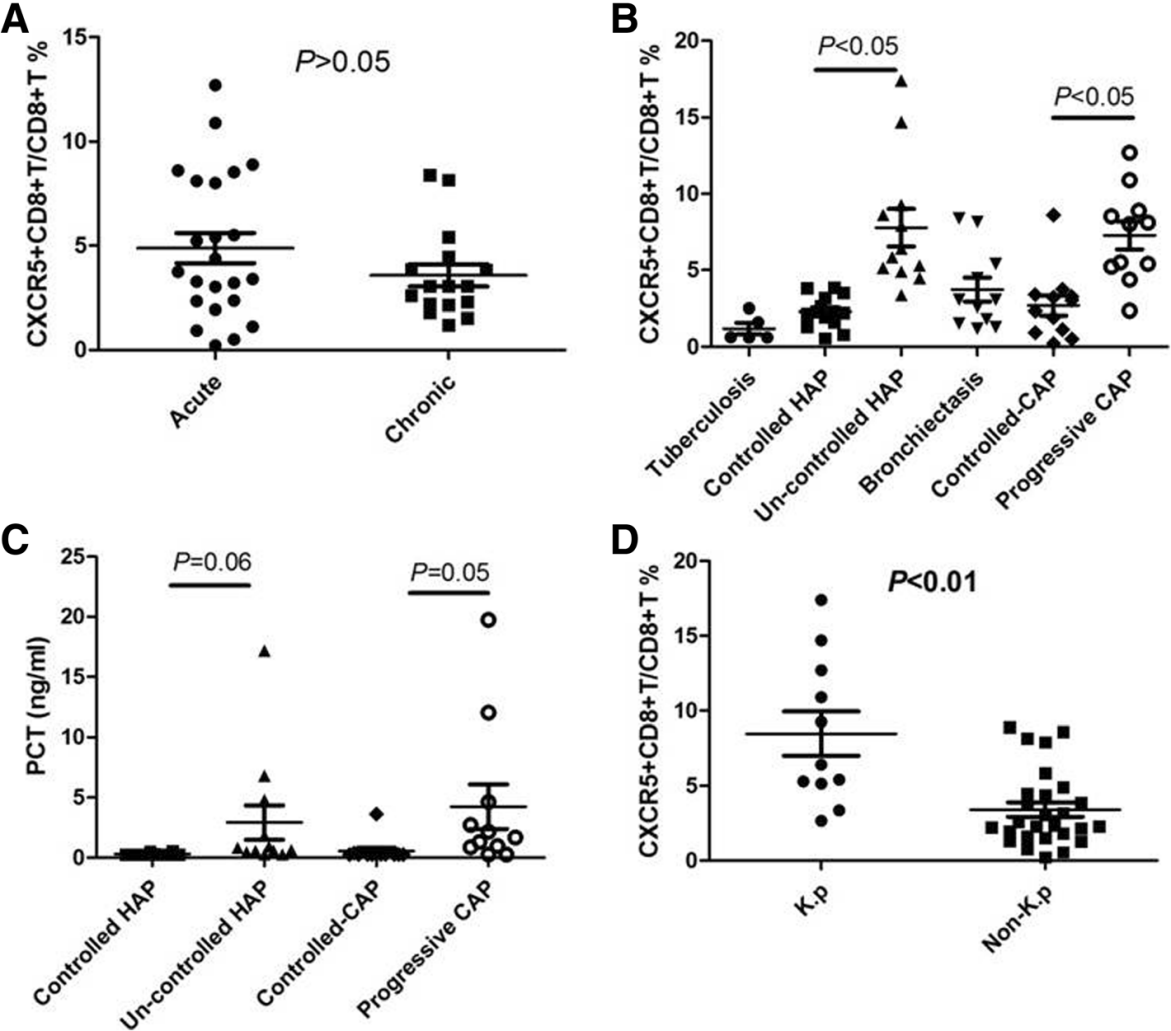
CXCR5 + CD8 + T cells are increased in the peripheral circulation in patients with uncontrolled CAP and progressive HAP compared controlled patient cohorts. Comparison of the circulating CXCR5 + CD8 + T cells and PCT between acute and chronic infection (**a**), among tuberculosis, bronchiectasis and community or hospital acquired pneumonia (**b**), between K.p and non-K.p infection (**c**, **d**). Results are presented as the mean with SD. Levels were compared by Student’s t-test

To do this, we selected patients with CAP (resulting in acute infection), bronchiectasis (*n* = 11) and chronic airway-open associated respiratory tract infection (resulting in chronic infection, *n* = 5). By flow cytometry, we characterized the number of circulating CXCR5-expressing CD8+ T cells in all individuals (Fig. 1). Strikingly, we did not visualize a substantial accumulation of circulating CXCR5^+^CD8^+^ T cells in chronically infected cases compared to chronically infected cases (3.58% ± 0.54 vs 4.88% ± 0.73, *P* = 0.19, Fig. 2a). Furthermore, CXCR5 + CD8+ T cells were not detected in peripheral blood in *Mycobacterium tuberculosis* infected patients (1.19% ± 0.38, *n* = 5). Notable, as showed in Fig. 2b, during an exacerbation of CAP and HAP, there was a significant increase in circulating CXCR5^+^CD8^+^ T cells compared to disease-controlled CAP (7.27% ± 0.91 vs 2.69% ± 0.64, *P* < 0.05) and HAP (7.77% ± 1.23 vs 2.30% ± 0.28, *P* < 0.05), respectively. Of note was the finding that PCT was weakly correlated with severity of respiratory infections (Fig. 2c).

### Pathogenic bacteria and circulating CXCR5^+^ CD8+ T-cells

A total of 38 pathogenic bacteria were detected in 65 patients, of which 27 patients were infected with Gram negative bacteria, 5 patients were infected with Gram positive bacteria, 6 patients were infected with fungal. Patients in Gram negative group were mainly infected with Klebsiella pneumonia (K.p, *n* = 11), *Pseudomonas aeruginosa* (*n* = 6), Acinetobacter baumannii (*n* = 4), while patients in Gram positive group were mainly infected with *Staphylococcus aureus* (n = 4). We further calculated the ratio of CXCR5 + CD8+ T cells/CD8+ cells to decide if these cells were relatively increased or decreased in special pathogenic bacteria-infected patients. Interestingly, we found that the circulating CXCR5^+^ CD8+ T-cells were significantly elevated in K.p-infected subjects compared to non-K.p-infected subjects (8.48% ± 1.47 vs 3.41% ± 0.48, *P* < 0.05, Fig. 2d).

### Circulating CXCR5^+^CD8^+^ T-cells are associated with evaluated PCT level

**Fig. 3:**
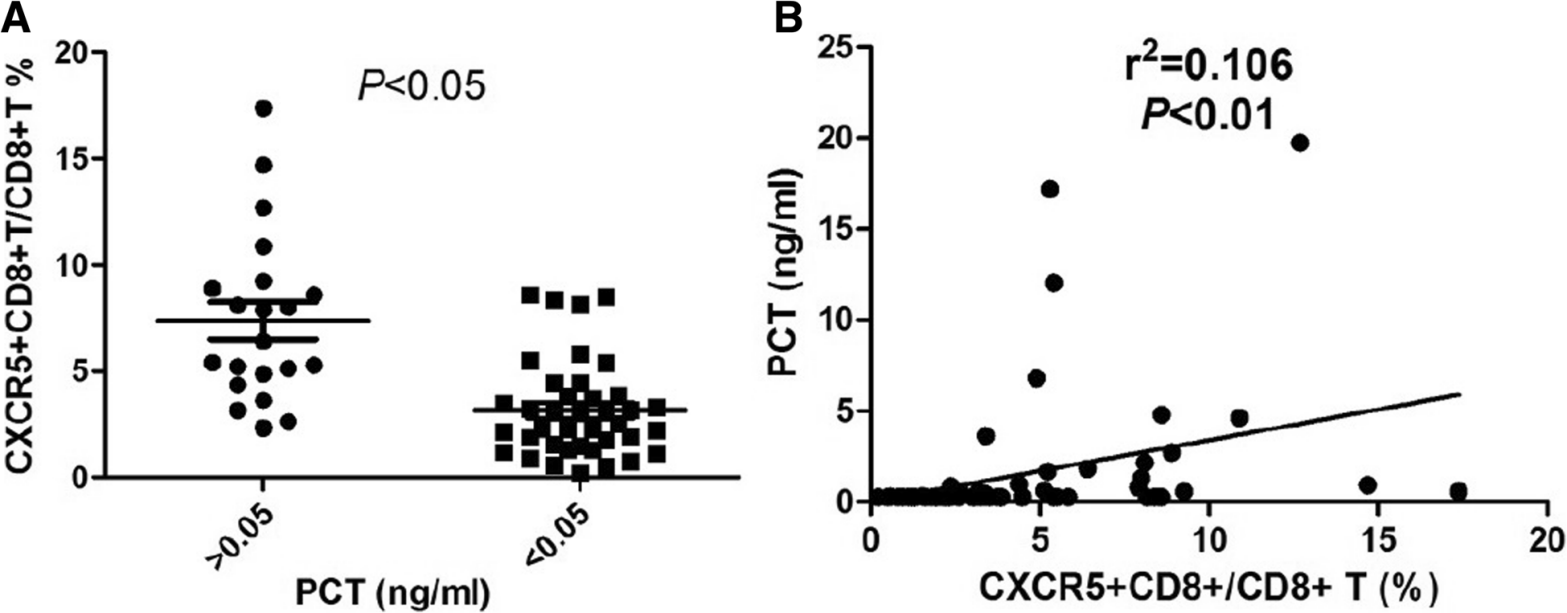
Elevation of PCT was positively correlated with increased CXCR5 + CD8+ T cells. **a**, Levels of circulating CXCR5 + CD8 + T cells between patients with PCT > 0.05 and PCT < 0.05 ng/ml. **b**, Scatterplot of CXCR5 + CD8+ T cell vs PCT. Each point in the scatter plot represents the value of two variables for a given observation. The rank Spearman correlation coefficient confirms that ratio of CXCR5 + CD8+ T cells and PCT are correlated

PCT is an acute-phase protein released into the bloodstream mainly in response to bacterial infection. To explore the association of circulating CXCR5-expressing T-cells with the severity of bacterial infection, we determined the correlation between CXCR5 + CD8 + T-cells and serum PCT concentration. As shown in Fig. 3, frequencies of circulating CXCR5^+^ CD8+ T-cells were higher in patients with PCT > 0.5 ng/ml than these in patients with PCT < 0.5 ng/ml (7.38% ± 0.87 vs 3.18% ± 0.36, *P* < 0.05). Correlations between PCT and CXCR5 + CD8 + T-cells were also identified (r^2^ = 0.11, *P* = 0.01). Accordingly, a positive correlation was found between the frequencies of CXCR5 + CD8+ T cells with PCT level in these individuals.

### Circulating CXCR5^+^CD8^+^ T-cells and diagnosis exacerbation of CAP and HAP

**Fig. 4:**
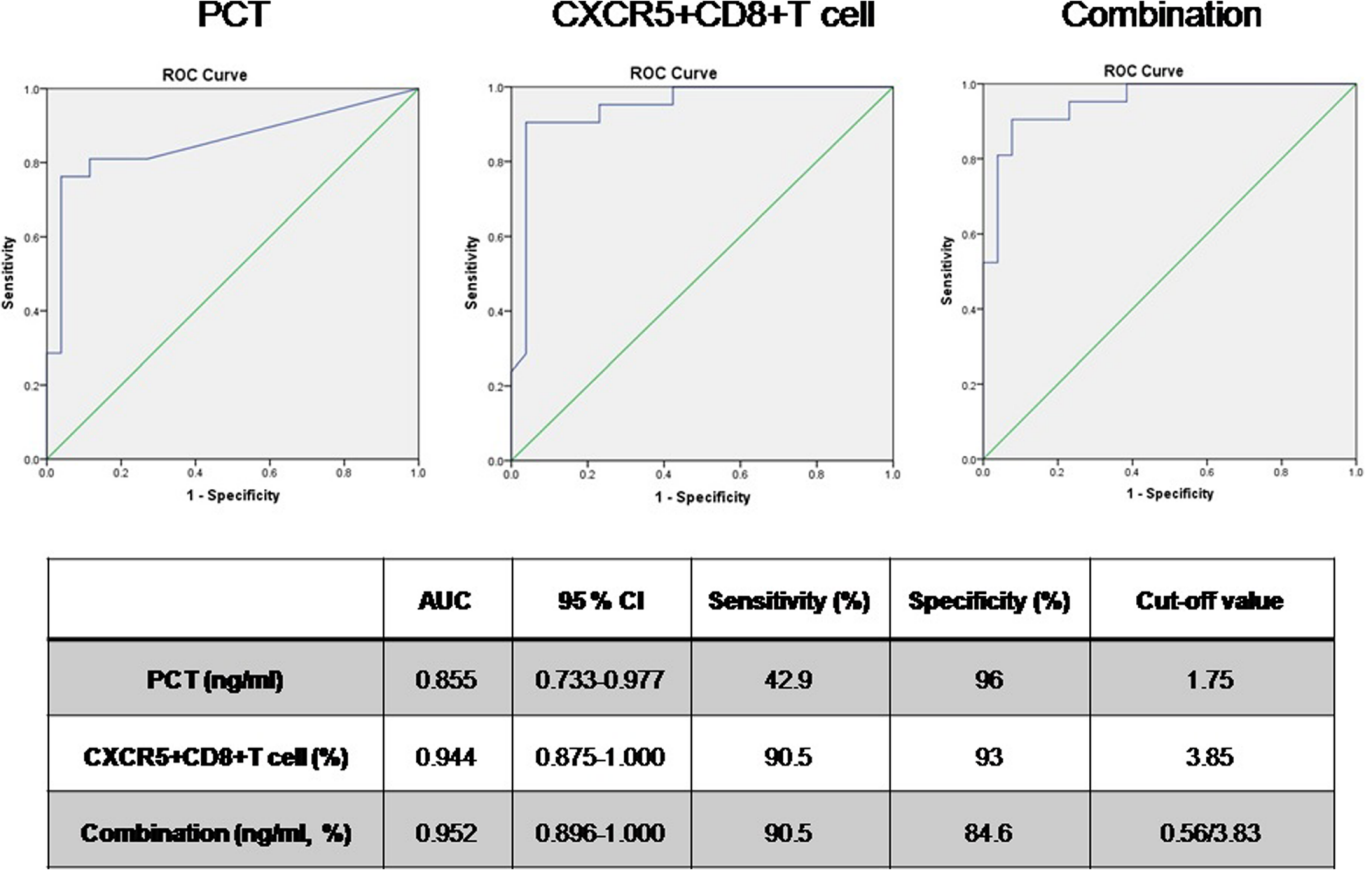
CXCR5 + CD8+ T cells could have diagnostic value when assessing the severity of bacterial respiratory infections. The optimal PCT, ratio of CXCR5 + CD8 + T cell and combination indicating exacerbation of CAP and HAP was analyzed

We next examine whether the circulating CXCR5^+^CD8^+^ T-cells and PCT is associated with the occurrence of exacerbation of CAP and HAP. As showed in Fig. 4, the area under the PCT curve was 0.855 (*P* < 0.05, 95% CI: 0.733–0.977) with sensitivity and specificity of 42.9 and 96%, respectively, when the critical value was 1.75 ng/ml. The area under the ratio of CXCR5 + CD8+ T cells/CD8+ cells curve was 0.944 (*P* < 0.05, 95% CI: 0.875–1.000) with sensitivity and specificity of 90.5 and 93%, respectively, when the critical value was 3.85%. Furthermore, the combination of PCT and ratio of CXCR5 + CD8+ T cells/CD8+ cells indicating exacerbation of disease was also analyzed. The sensitivity was found to be 90.5%, specificity was found to be 84.6%, and the area under the ROC curves was 0.952 (95% CI: 0.896–1.000). It was suggested that the determination of CXCR5-expressing CD8+ T-cells alone can show good results for the clinical prediction of exacerbation of CAP or HAP.

### Expression of surface markers by CXCR5 + CD8+ T cells

**Fig. 5:**
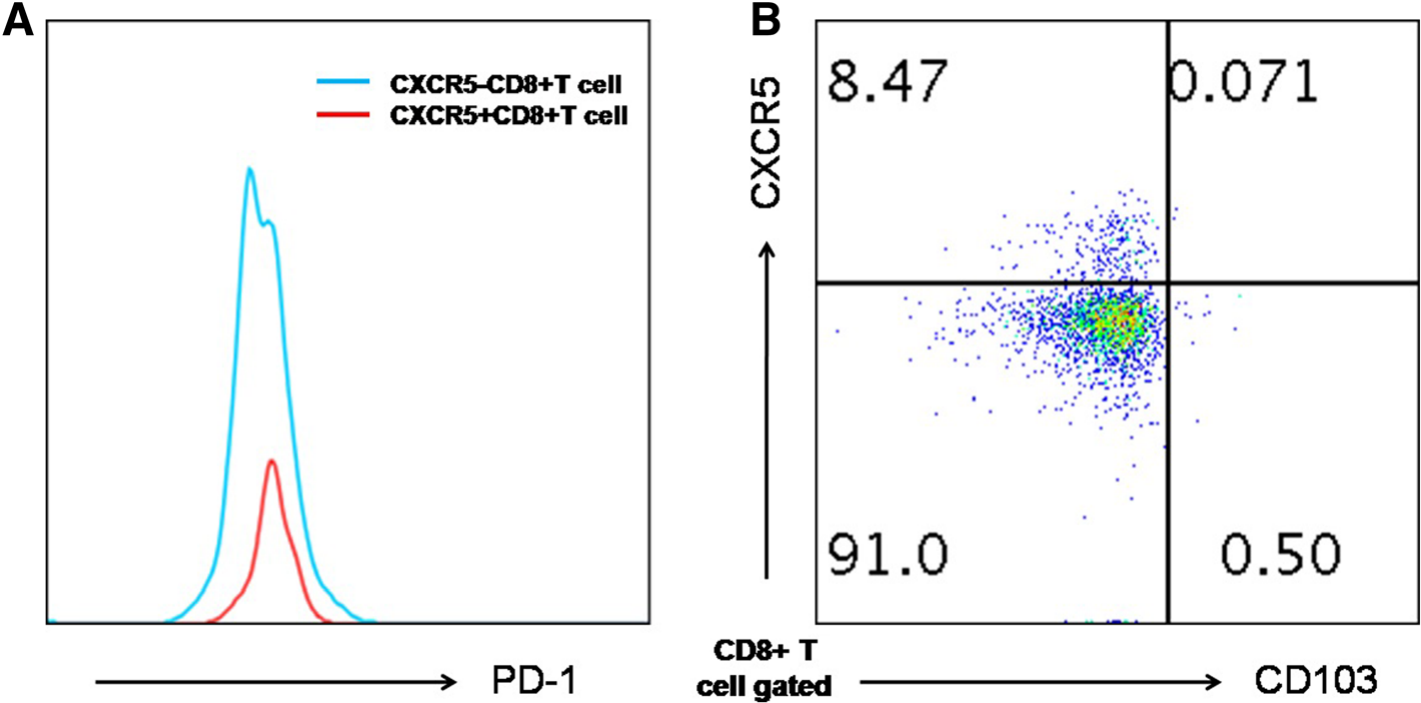
The phenotype of CXCR5+ and CXCR5-CD8+ T cells. Circulating CXCR5-expressing CD8+ T-cells had a comparable level of PD-1 compared to their CXCR5- counterparts (**a**), exhibited low or null expression of the CD103 (**b**). Representative FACS plots are shown

Finally, we compared the phenotype of CXCR5+ and CXCR5-CD8+ T cells. Circulating CXCR5-expressing CD8+ T-cells exhibited low or null expression of the CD103 and had a comparable level of PD-1 compared to their CXCR5- counterparts (Fig. 5). Furthermore, in all pneumonia individuals, no detectable of CD103 + CD8 + T cells were seen in peripheral blood (data not show).

## Discussion

Compared to T follicular helper cells (TFH) cells, current research on the CXCR5 + CD8+ T cells is relatively scarce. Several studies suggest that CXCR5+ CD8+ T cells represent a subset of follicular cytotoxic CD8+ T cells and may contribute to virus control in B cell follicles [2, 3]. Some CXCR5-CD8+ T cells with the ability to contain lymphocytic choriomeningitis virus (LCMV) have been found in germinal center (GC) in mice and in blood of HIV-infected patients, where their levels correlated with viral load. In patients with HIV, the number of virus-specific CXCR5 + CD8+ T cell subset is inversely correlated with viral load in LNs [5]. Peripheral and GC CXCR5-CD8+ T cells are also present in SIV-infected macaques, where their levels increase after immunization, and it is higher in macaques controlling infection than ones who do not. CD8+ T cells can still contain viral replication in chronic infections although the mechanism of this containment is largely unknown [10, 11].

In this study, we aimed to determine if CXCR5 + CD8+ T cell was a valuable biomarker for bacterial infection in subjects with pneumonia. As expected, CXCR5+ CD8+ T cells are present in human peripheral blood and therefore can be readily quantified. As reported previously, the novel population of CXCR5+ cell was only generated in the chronically infected mice, showing that antigen persistence drives the generation of this novel CD8+ T cell subset [2, 3]. Strikingly, we did not visualize a substantial accumulation of circulating CXCR5^+^CD8^+^ T cells in chronically infected cases. However, patients on progressive CAP and HAP had higher frequencies of CXCR5 + CD8 + T cell compared with controlled CAP and HAP, respectively. These results suggest a redistribution of this subset occur after the bacterial exacerbation. One possibility is that the bacterial antigens within lung tissues result in the increasing frequency of CXCR5+ CD8 + T cell in blood.

PCT is a kind of protein mainly produced by thyroid C-cells, which stays at a low level under normal body conditions. Several studies have suggested that PCT is the most useful biomarker for diagnosis of sepsis, with precedence over other laboratory indicators [12, 13]. In this study, from analysis of ROC curves, CXCR5 + CD8+ T cells can alone act as a stronger predictive index of bacterial exacerbation when the PCT possess lower sensitivity to clinical prediction of exacerbation of CAP or HAP. With the advantages of simple and effective detection, it is of great significance for avoiding missed diagnosis in patients with bacterial exacerbation and providing guidance for appropriate control.

Lung infections caused by carbapenem-non-susceptible *Klebsiella pneumoniae* constitute a worldwide problem associated with high rates of treatment failure and mortality [14, 15]. In our collection of isolates, Klebsiella pneumonia infected-rate mounted to 16.9%. Elevated levels of CXCR5 + CD8+ T cells in the *Klebsiella pneumoniae* infected-group were markedly higher than these in the others group. Given the severity of *Klebsiella pneumoniae* infections and dependence of generation of CXCR5 + CD8 + T cell on antigen persistence, these cells are associated with developing immune response against highly pathogenic *Klebsiella pneumoniae*.

It was reported that circulating HIV-specific CXCR5 + CD8+ T-cells had a higher production of IL-21 than CXCR5- cells [5]. And follicular cytotoxic CD8+ T cells express granzyme A and B and perforin at higher levels than their CXCR5- counterpart [16]. We also characterized the function state of circulating CXCR5-expressing T-cells by the expression of PD-1. In our study, we identified circulating CXCR5 + CD8+ T-cells and CXCR5-CD8+ T-cells, which exhibited comparable level of PD-1. It has been indicated that virus-specific CD8+ T cells present in GCs of humans and macaques may not be enough to clear the increasing population of infected TFH cells. Taken together, the increase of CXCR5 + CD8 + T cell may be functionally impaired or exhausted.

Whether circulating CXCR5+ cells are the resident counterpart of CD8 + T cells has been controversial. Here, CXCR5 + CD8+ T cells did not be defined as TCM cells by virtue of negative CD103 expression [17, 18]. Then, these cells might provide new correlates of protection, disease progression, or treatment response, pointing toward potential therapeutic strategies.

There are still limitations about this research. The detailed mechanism of increased CXCR5 + CD8+ T cells need to be further researched. The function of these cells in bacterial infection should also be studied thoroughly in future.

## Conclusion

Taken together, the current study explored the diagnostic value of circulating CXCR5 + CD8+ T cells in bacterial respiratory infections. Overall, our studies on circulating CXCR5 + CD8+ T cells have shed light on the its important role in the pathogenesis process of uncontrolled respiratory bacterial infectious diseases. It was indicated that candidate bacterial vaccines should attempt to preferentially elicit these cells.

## Abbreviations

BALF: Bronchoalveolar lavage fluid
CAP: Community acquired pneumonia
CRP: C-reactive protein
CXCR5: C-X-C chemokine receptor type 5
FACS: Fluorescence activated cell sorter
GC: Germinal center
HAP: Hospital acquired pneumonia
K.p: Klebsiella pneumonia
LCMV: Lymphocytic choriomeningitis virus
LN: Lymph node
PCT: Procalcitonin
ROC: Receiver operating characteristic curve
TFC: T follicular helper cells
